# Advances and Challenges of Antibody Therapeutics for Severe Bronchial Asthma

**DOI:** 10.3390/ijms23010083

**Published:** 2021-12-22

**Authors:** Yuko Abe, Yasuhiko Suga, Kiyoharu Fukushima, Hayase Ohata, Takayuki Niitsu, Hiroshi Nabeshima, Yasuharu Nagahama, Hiroshi Kida, Atsushi Kumanogoh

**Affiliations:** 1 Department of Respiratory Medicine and Clinical Immunology, Osaka University Graduate School of Medicine, 2-2 Yamadaoka, Suita, Osaka 565-0871, Japan; y.abe@imed3.med.osaka-u.ac.jp (Y.A.); Yasu031055@imed3.med.osaka-u.ac.jp (Y.S.); haya.oohata@gmail.com (H.O.); mosatsu1987@gmail.com (T.N.); kumanogo@imed3.med.osaka-u.ac.jp (A.K.); 2 Laboratory of Host Defense, World Premier Institute Immunology Frontier Research Center (WPI-IFReC), Osaka University, Osaka 565-0871, Japan; h-nabeshima@ifrec.osaka-u.ac.jp (H.N.); y-nagahama@ifrec.osaka-u.ac.jp (Y.N.); 3 Department of Host Defense, Research Institute for Microbial Diseases (RIMD), Osaka University, Osaka 565-0871, Japan; 4 Department of Respiratory Medicine, National Hospital Organization, Osaka Toneyama Medical Centre, 5-1-1 Toneyama, Toyonaka, Osaka 560-0852, Japan; kida.hiroshi.sv@mail.hosp.go.jp; 5 Department of Immunopathology, World Premier Institute Immunology Frontier Research Center (WPI-IFReC), Osaka University, Osaka 565-0871, Japan; 6 Integrated Frontier Research for Medical Science Division, Institute for Open and Transdisciplinary Research Initiatives, Osaka University, Osaka 565-0871, Japan

**Keywords:** asthma, refractory asthma, antibody therapeutics, biomarker

## Abstract

Asthma is a disease that consists of three main components: airway inflammation, airway hyperresponsiveness, and airway remodeling. Persistent airway inflammation leads to the destruction and degeneration of normal airway tissues, resulting in thickening of the airway wall, decreased reversibility, and increased airway hyperresponsiveness. The progression of irreversible airway narrowing and the associated increase in airway hyperresponsiveness are major factors in severe asthma. This has led to the identification of effective pharmacological targets and the recognition of several biomarkers that enable a more personalized approach to asthma. However, the efficacies of current antibody therapeutics and biomarkers are still unsatisfactory in clinical practice. The establishment of an ideal phenotype classification that will predict the response of antibody treatment is urgently needed. Here, we review recent advancements in antibody therapeutics and novel findings related to the disease process for severe asthma.

## 1. Introduction

Asthma is a disorder ordinarily characterized by allergic chronic airway inflammation. Usually, this condition is sensitive to corticosteroids and the widespread use of inhaled corticosteroids (ICS) has markedly reduced asthma emergencies. However, 5–10% of asthma patients are refractory to the maximum combination treatment of high-dose ICS, long-acting β_2_-agonists, and long-acting muscarinic antagonists. In 2014, the American Thoracic Society and European Respiratory Society published guidelines defining severe asthma as a condition that requires treatment with high-dose ICS, plus other long-term control medications (and/or oral corticosteroids (OCS)), or is poorly controlled regardless of these treatments [1]. These guidelines indicate that a diagnosis of severe asthma requires a correct diagnosis of asthma, confirmation of the presence of comorbidities (sinus disease, obesity, aspirin asthma, chronic obstructive pulmonary disease), and appropriate assessment of asthma control.

Newly developed antibody therapies targeting cytokines involved in the pathophysiological process of asthma have led to reduced exacerbations and improved symptom control and lung function in a subgroup of severe asthmatics. However, the efficacies of current antibody therapeutics are still unsatisfactory in clinical practice. This is, in part, because of the heterogeneity of severe asthma. Here, we review recent advancements in antibody therapeutics and novel findings related to the disease process of severe asthma.

## 2. Current Understanding of Severe Bronchial Asthma

**Figure 1:**
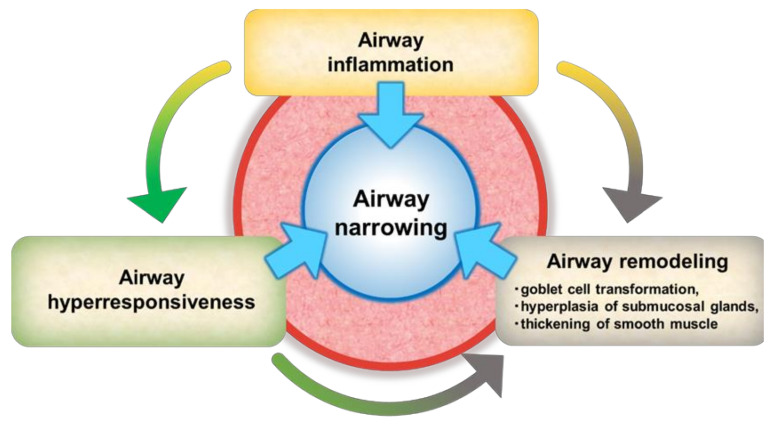
Mechanisms of the onset and exacerbation of severe asthma.

Asthma is a disease characterized by chronic airway inflammation and usually reversible airflow limitation. However, uncontrolled disease activity leads to sustained airway inflammation, and increased airway hyperresponsiveness (AHR) and airway remodeling, resulting in persistent respiratory symptoms. Airway inflammation, airway remodeling, and airway hyperresponsiveness differentially contribute to the clinical features of each patient with severe asthma [2] (Figure 1). Typically, airway inflammation is a triggering/exacerbation factor for airway hypersensitivity and airway remodeling, but the interaction of each of the three factors becomes a further exacerbation factor as asthma progresses. Therefore, treatment strategies targeting airway inflammation as well as airway hyperresponsiveness and airway remodeling would be more effective than targeting only airway inflammation.

Chronic airway inflammation in asthma is typically characterized by eosinophil infiltration, overproduction of IgE, and Th2 cytokines, including IL-4, IL-5, and IL-13. IL-5, which is essential for eosinophil activation and proliferation and is derived from Th2 cells and Group 2 innate lymphoid cells (ILC2s). IL-4 and IL-13, derived from Th2 cells, promote the production of specific IgE antibodies from B cells. IgE antibodies bind to the IgE receptor (FcεRI) on the surface of mast cells and prepare them for activation during allergen exposure. IL-13, secreted from Th2 cells and ILC2s, shares the IL-4 receptor and induces smooth muscle hyperplasia, goblet cell hyperplasia, and mucous secretion, leading to airway remodeling and decreased respiratory function. Lung ILC2s respond to the alarmin IL-33, or IL-25 and TSLP released by epithelial cells, which induce their activation and the production of large amounts of IL-5 and IL-13. Thus, IgE, IL-5, and IL-4/IL-13 are particularly important in the pathogenesis of type 2 asthma. Blood eosinophils have been proposed as a surrogate marker of airway eosinophilia [3]. Elevated numbers of blood eosinophils have been associated with more severe asthma and have shown to predict a higher risk of asthma exacerbation [4]. Measurement of FeNO is another non-invasive way to quantify Th2 high airway inflammation. Nitric oxide (NO) is produced by epithelial cells lining the airways. Inducible nitric oxide synthase (iNOS) is induced by Th2 type inflammation, where it is largely driven by IL-4 and IL-13, leading to the increased production of NO. Similar to elevated blood eosinophils, elevated FeNO is predictive of asthma exacerbations and asthma severity [5,6]. In addition, simultaneously increased FeNO and blood eosinophils were associated with a higher likelihood of AHR [6]. Because Th2 cytokines, including IL-13 and IL-4, act on goblet cell hyperplasia, fibroblast-to-myofibroblast transformation, collagen deposition, and airway smooth muscle contraction [7], high FeNO levels in patients with severe asthma may be affected by airway remodeling. In another study involving 310 young adult subjects with suspected cough variant asthma, FeNO levels and the eosinophil count percentage in induced sputum, in addition to the Forced expiratory flow at 25–75% of FVC (FEF25-75), were associated with AHR [8]. Of note, FeNO can be affected by many external variables including ambient air quality, smoking, sinus disease, allergic rhinitis, and virus infection. These factors need to be considered when interpreting results. As already described, IgE is a key factor in high Th2 inflammation. Total serum IgE and specific IgE are the most common risk factors for allergic asthma. In a cluster analysis by the National Institute of Health/National Heart, Lung, and Blood Institute Severe Asthma Research Program pediatric cohort, children with severe asthma had higher serum IgE levels and increased sensitization to aeroallergens [9].

**Figure 2:**
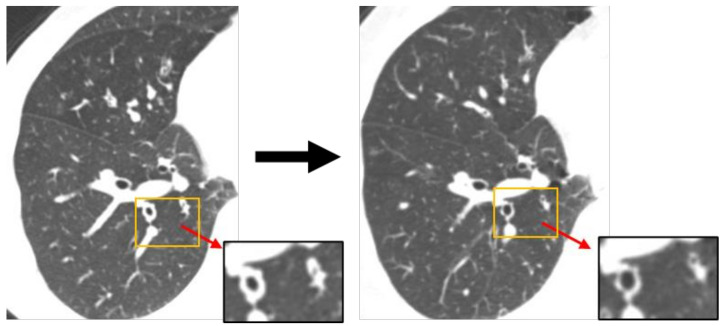
Chest CT before and one year after the start of dupilumab.

Airway remodeling describes structural changes of the airway wall, including fibrosis, airway smooth muscle hypertrophy, and goblet cell hyperplasia. Among these, the molecular mechanism of goblet cell hyperplasia has been intensively studied. SAM (sterile α-motif) pointed domain containing ETS transcription factor (SPDEF) and forkhead box protein (Foxa3) are the main transcription factors that regulate differentiation into goblet cell hyperplasia [10]. These transcription factors are induced by Th2 cytokines in the airway epithelium and promote thymic stromal lymphopoietin (TSLP) production in airway epithelial cells, which exacerbates Th2 inflammation [11]. Epithelial-mesenchymal transition (EMT), and the migration and proliferation of cultured airway smooth muscle cells have been used as surrogate experimental models to investigate the molecular mechanisms involved in fibrosis and airway smooth muscle hypertrophy. By using these models, transforming growth factor (TGF)-β was shown to be an important factor in the airway remodeling in asthma [12,13,14,15]. Clinically, the standard assessment of remodeling is obtained by a biopsy of the lungs and airways by surgery or bronchoscopy. However, bronchial biopsy is invasive and not applicable to routine clinical settings. It also requires expert knowledge, therefore, indirect analytical methods using remodeling markers in the blood, urine, and sputum have also been developed [16]. Periostin is a matricellular protein secreted by bronchial epithelial cells and lung fibroblasts in response to the Th2 cytokines, IL-13, and IL-4 [17]. Periostin-high asthma patients had clinical characteristics including eosinophilia, high FeNO, aspirin intolerance, nasal disorders, and late-onset disease [18]. In addition, periostin was reported to be associated with hyporesponsiveness to ICS. Other alternatives, such as high-resolution computed tomography, endobronchial ultrasonography, and lung function measurements, can also be used as screening tools in clinical practice [19]. Computed tomography, a non-invasive process, allows the study of the airway lumen and wall dimensions, which might help evaluate airway remodeling in children and clinical studies [20]. This approach can be used to identify the airway tree and evaluate changes in remodeling after treatment, as well as determine air trapping. Endobronchial ultrasound (EBUS) is performed with an ultrasonographic probe through the working channel of a fiberoptic bronchoscope. It can access airways 4 mm in internal diameter and visualize multiple layers of the airway wall without the use of radiation [21]. Decreased values of V50 and V25 and increased values of the V50/V25 ratio are useful for the early detection of peripheral airway diseases [22]. Because persistent airflow obstruction is caused by airway remodeling, V50/V25, which indicates a peripheral airflow obstruction, can also be an indicator of airway remodeling. In our case of severe asthma, airway remodeling was diagnosed by low FEV1% (62.79%), highV50/V25 (5.48), and thickening of the airway wall assessed by CT. In this case, after one-year treatment by dupilumab, FEV1 (2.43 L → 3.48 L) was increased, and V50/25 (5.48 → 4.23) was decreased. Furthermore, the airway wall thickness was attenuated (Figure 2), which strongly suggests the improvement of airway remodeling by dupilumab.

AHR is defined as the increased sensitivity and enhanced narrowing of the airways in response to physical or chemical stimuli. AHR is caused by abnormalities in the airway smooth muscle, which is primed by Th2 inflammation. Recent studies suggested that IL-13 and IL-4 signaling through the IL-4 receptor is responsible for these abnormalities, by upregulating the expressions of histamine receptor H1 (HRH1) and cysteinyl leukotriene receptor 1 (CYSLTR1) in airway smooth muscle cells [23]. Clinically, bronchial provocation tests (BPTs) are used to measure AHR, in which airway constrictors such as methacholine or cAMP are inhaled and their propensities to develop airflow obstruction are examined. BPTs are the gold standard test, but their methodology is complicated for general clinical practice, time consuming, and can induce severe bronchospasms. Therefore, alternative markers that reflect AHR have been explored. Kono et al. investigated a correlation between airway hyperresponsiveness measured by BPTs and the variables obtained by spirometry tests and reported that the FEF25-75% predicted showed the highest correlation with airway hyperresponsiveness [24]. Respiratory system resistance (Rrs) measured by the forced oscillation technique at 5 Hz (R5) and 20 Hz (R20) is a marker of airway caliber. A larger R5 reflects small airway dysfunction and was reported to be associated in part with airway hypersensitivity [25].

As described above, airway inflammation is often a triggering/exacerbation factor for airway hypersensitivity and airway remodeling, but the interaction of each of the three factors becomes a further exacerbation factor as asthma progresses. Therefore, monoclonal antibodies targeting airway inflammation as well as airway hyperresponsiveness and airway remodeling would be more effective than monoclonal antibodies targeting only airway inflammation.

## 3. Therapeutic Antibodies for Bronchial Asthma

There are four types of monoclonal antibodies available to treat bronchial asthma, including anti-IgE antibody, anti-IL-5 antibody, anti-IL-5 receptor α antibody, and anti-IL-4 receptor α antibody (Table 1).

Omalizumab, a humanized monoclonal IgG1κ antibody to IgE, inhibits the binding of IgE to high-affinity receptor, FcεRI, on mast cells or basophils and low-affinity receptor, FcεRII, on B cells, T cells, Langerhans cells, macrophages, monocytes, eosinophils, and platelets. Upon the cross-linking of membrane bound IgE by a specific allergen, mast cells or basophils degranulate and secrete mediators such as histamine, which induce bronchoconstriction in asthmatic patients. Omalizumab does not bind to IgE, which binds to its high-affinity receptor IgεRI, suggesting that there is no risk of the cross-linking of membrane-bound IgE [26]. Because FcεRI and FcεRII are stabilized by the binding of IgE, the administration of omalizumab reduces the expression of these receptors on inflammatory cells. Recently, it was reported that bronchial epithelial cells and airway smooth muscle cells from patients with bronchial asthma also expressed FcεRI and FcεRII [27]. An ex vivo study investigating the impact of omalizumab on specific and nonspecific AHR in proximal and distal human airways passively sensitized with serum from asthmatic donors showed it significantly suppressed the contractile response [28]. Therefore, omalizumab might directly affect the three components of asthma: airway inflammation, airway remodeling, and AHR.

Mepolizumab and reslizumab are humanized monoclonal IgG1κ and IgG4 antibodies, respectively, that bind with high affinity to human IL-5, thus preventing its interaction with the α subunit of the IL-5 receptor [29]. In patients with asthma, IL-5 is locally produced by Th2 lymphocytes, group 2 innate lymphoid cells (ILC2s), and epithelial cells in the airway mucosa. IL-5 stimulates the differentiation of eosinophils in the bone marrow and mobilization of eosinophils from the bone marrow. IL-5 also acts on basophils and stimulates the release of mediators, including histamine and leukotrienes. Therefore, mepolizumab and reslizumab inhibit airway inflammation in asthmatic patients. Although IL-5-deficient mice showed a reduction of airway remodeling in an ovalbumin-induced allergic airway inflammation model, the authors discussed that airway remodeling was caused by TGF-β produced by eosinophils and that the effect of IL-5 on airway remodeling was indirect [30].

A different mechanism of function characterizes the biological targeting of the IL-5 cascade. Benralizumab is a humanized IgG1κ monoclonal antibody, which binds to IL-5Rα. The IL-5 receptor is a heterodimer composed of IL-5-specific IL-5Rα and β subunits, which is commonly used by IL-5, IL-3, and GM-CSF. Benralizumab triggered apoptosis in eosinophils and basophils by antibody-dependent cell-mediated cytotoxicity (ADCC) associated with natural killer cells, a mechanism potentiated by afucosylation [31,32]. This effect is expected to reduce eosinophil counts, even in the presence of eosinophil activators such as IL-5, resulting in the rapid loss of peripheral blood eosinophils. Therefore, it can reduce eosinophilic airway inflammation. Ex vivo experiments demonstrated benralizumab suppressed AHR induced by histamine administered when significantly high cAMP levels were present, and that its effect was greater compared with mepolizumab [33]. This suggested that the improvement in the concentration of cAMP by inhibiting the IL-5/IL-5Rα pathway may converge to prevent AHR. An in vitro study using human ASM cells confirmed the beneficial role of benralizumab in reversing airway remodeling [34].

Dupilumab is a humanized IgG1k monoclonal antibody to the IL-4 receptor α subunit (IL-4Rα), common to both IL-4R complexes: type I (IL-4Rα/γc; IL-4 specific) and type II (IL-4Rα/IL-13Rα1; IL-4 and IL-13 specific). The type I IL-4R complex is expressed on hematopoietic cells and the type II IL-4R complex is expressed on hematopoietic and non-hematopoietic cells including epithelial cells and fibroblasts. In experimental mouse models of bronchial asthma using IL-4Rα-knockout mice and IL-13Rα1-knockout mice, the type I IL-4R complex is thought to activate Th2 inflammation, whereas the type II IL-4R complex inhibits Th2 inflammation but augments AHR and airway remodeling [35]. Although the precise mechanisms have not been reported, dupilumab is thought to function as a dual receptor antagonist of the type I and type II IL-4R complexes, by inhibiting their biological actions [36,37].

## 4. Clinical Effects of Antibodies for Bronchial Asthma Patients

### 4.1. Omalizumab

Randomized controlled trials (RCTs) and a systematic review indicated that omalizumab reduced asthma exacerbation and OCS intake, improved quality of life, and contributed to symptom control [42,43,51]. Omalizumab also has a good safety profile. An RCT reported that omalizumab improved AHR measured by cAMP after 4 weeks of treatment [38]. In real-world studies and a systematic review, omalizumab reduced asthma exacerbation, contributed to the step-down of asthma treatment, and improved the quality of life in long-term users without compromising safety [58,59]. Since omalizumab was first licensed as a therapeutic antibody for bronchial asthma two decades ago, some studies have addressed omalizumab discontinuation after long-term treatment. Two RCTs and one real-world study showed that a proportion of patients could withdraw from long-term omalizumab treatment without relapse [60,61,62].

### 4.2. Mepolizumab

RCTs confirmed that mepolizumab reduced asthma exacerbation, emergency department visits, and hospitalization [44]. These studies also demonstrated that mepolizumab improved health-related quality of life (HRQOL) among patients with severe asthma [63]. In real-world settings, treatment with mepolizumab reduced asthma exacerbation, OCS requirement, and the rescue use of short-acting **β**-agonists, resulting in the step-down of maintenance therapy of asthma [53]. It also improved the HRQOL. Regarding its effectiveness for AHR, an RCT showed that mepolizumab had no significant effect on AHR measured by BPT using methacholine [64].

### 4.3. Reslizumab

RCTs reported that reslizumab reduced asthma exacerbation and improved lung function, symptoms, and HRQOL [45,65,66]. A real-world study supported the results of the RCTs showing reslizumab reduced asthma exacerbations, maintenance OCS use, and health-care resource use. Reslizumab was also shown to improve asthma symptoms and lung function [67]. Although mepolizumab is an IgG1**κ** antibody and reslizumab is an IgG4 antibody, both reduced asthma exacerbation and improved the FEV1 [45].

### 4.4. Benralizumab

RCTs and a systematic review reported that benralizumab was effective at reducing asthma exacerbations, improving prebronchodilator FEV_1_, asthma symptoms, HRQOL, and reducing OCS intake in patients with severe asthma [49,52,68,69]. Real-world studies re-confirmed these clinical benefits. Benralizumab reduced asthma exacerbations and OCS dose, and improved HRQOL [70]. Despite the improvement in AHR in an ex vivo model [33], a clinical trial reported benralizumab did not significantly improve AHR measured by BPT using histamines [71]. Regarding its effectiveness on airway remodeling, benralizumab reduced airway smooth muscle mass using a computational modeling approach [34].

### 4.5. Dupilumab

RCTs reported that dupilumab decreased asthma exacerbations and improved pre-bronchodilator FEV_1_ and asthma symptoms [55,72]. Unlike other therapeutic antibodies, the RCTs included patients with moderate disease, making dupilumab available for patients with moderate asthma. Real-world data supported the results of the RCTs, showing that dupilumab reduced asthma exacerbations, the daily dose of OCS, and FeNO levels, and improved asthma symptoms [73,74].

## 5. Clinical Predictors of Good Responders to Each Antibody Therapy

As described above, currently approved monoclonal antibodies have been shown to have many clinical effects. However, these treatments are not successful in all patients, and super-responders to specific therapeutic antibodies have been reported. Therefore, studies investigating clinical predictors of good responders to each antibody therapy have been initiated.

In patients treated with omalizumab, the baseline total IgE does not predict likelihood of response, allergen-specific IgE, or the reduction of total IgE 4-weeks after the initiation of omalizumab might predict response [75]. In a real-world study, each of the three Th2-inflammation markers, including FeNO, peripheral blood eosinophil, and serum periostin, or their combinations, predicted response to omalizumab [37]. The biomarkers for withdrawal of mepolizumab also have been proposed. The downregulation of basophil allergen sensitivity (CD-sens) and regulatory T cells were reported to be the candidates for the cessation criteria [76,77]

In an RCT of mepolizumab, high blood eosinophil counts predicted a good response [44]. Although a post hoc analysis of two RCTs could not identify the additional baseline characteristics associated with a response to mepolizumab [78], a retrospective review of patients who received at least 16 weeks of treatment with mepolizumab showed that the presence of nasal polyposis, a lower BMI, and a significantly lower prednisolone dose at baseline might predict a good response to mepolizumab [46]. Another study following patients receiving anti-IL-5 treatment for two years, showed that adult onset, absence of nasal polyposis, FEV1 ≥ 80% predicted, asthma duration < 10 years, and BMI < 25 were baseline characteristics that predicted a super-response to anti-IL-5 treatment [79]. It was also reported that the responders to mepolizumab had a significantly lower level of CCL4/MIP-1β at baseline compared with non-responders [47].

RCTs of benralizumab showed that high blood eosinophil counts predicted a good response [49,52]. A post hoc analysis of two RCTs showed that nasal polyposis, pre-bronchodilator FVC < 65% of predicted, and age at diagnosis ≥ 18 years were the most important factors that influenced benralizumab responsiveness for improving lung function [50]. A real-world study reported a strongly eosinophilic phenotype, high blood eosinophil count, and elevated FeNO levels, as well as less severe disease were associated with super-responders [70]. Exploratory studies reported that low baseline levels of serum inflammatory cytokines and a serum miRNA response 8 weeks after the initiation of benralizumab were potential predictors of a good response [80,81].

The treatment effects of dupilumab were greater in patients with elevated Th2 biomarkers at baseline (blood eosinophils (≥150/uL) or FeNO (≥25 ppb)) [55].

## 6. Prospects for Severe Asthma Treatment

New potential targets for asthma treatments are being investigated globally. Alarmins including thymic stromal lymphopoietin (TSLP), IL-25, and IL-33, have an important role in T2-high asthma [82].

TSLP induces the strong activation of dendritic cells (DCs) [83], and DCs stimulated by TSLP drive naïve Th lymphocytes towards differentiation into active T2 cells producing IL-4, IL-5, and IL-13 [84]. TSLP also stimulates basophils, mast cells, and ILC2 [82,85,86]. Recent clinical trials have shown the effectiveness of anti-TSLP antibody for asthma [87]. TSLP is also a key airway remodeling mediator that promotes airway smooth muscle cells to increase airway smooth muscle mass migration [88]. TSLP might promote asthmatic airway remodeling by activating the p38 MAPK-STAT3 axis [89]. Tezepelumab is an anti-TSLP human monoclonal antibody that binds to TSLP and prevents TSLP binding to its receptor complex [90]. The first study of tezepelumab was conducted in patients with mild allergic asthma [91]. In that study, tezepelumab minimized the allergen-induced decline in the FEV1. It also reduced the level of post-allergen blood/sputum eosinophils and FeNO levels. In a phase IIb study of 584 adult patients, tezepelumab decreased the asthma exacerbation rate by 60–70% per year and improved the FEV1 without the use of bronchodilators, regardless of blood eosinophil numbers [92]. That study confirmed the ability of tezepelumab to suppress serum IgE concentrations, blood eosinophil counts, and FeNO levels. The safety and efficacy of tezepelumab to decrease airway inflammation and OCS intake are now being evaluated in phase II and III trials [82]. Tezepelumab is also expected to be effective in airway remodeling, but at present, there are no available studies of its in vitro or in vivo effects on airway remodeling. An RCT showed that tezepelumab induced a numerical improvement in the provoking dose of mannitol causing a 15% reduction in FEV1 (PD15) compared with placebo, and at the end of the treatment period, the proportion of patients without AHR to mannitol was significantly (*p* < 0.05) higher in the tezepelumab group than in the placebo group [93].

Although IL-25 has a pathogenic role in allergic inflammation, there has been no clinical study of anti-IL-25 monoclonal antibodies for the treatment of severe asthma.

IL-33 activates Th2 and group 2 innate lymphoid cells and induces allergic diseases including allergic rhinitis, spontaneous dermatitis, and asthma [94,95]. IL-33 stimulates ILC2 and mast cells to release IL-13 and induces airway hyperresponsiveness [96,97]. A phase II trial reported that an anti-IL-33 monoclonal antibody, REGN3500, improved the QOL of patients and controlled the symptoms of severe asthma [82].

Another potential target molecule is IL-13 and IL-13 blockade therapies, which are being investigated in clinical trials. Tralokinumab and lebrikizumab are mAbs that target IL-13 [98,99]. Phase 2 studies reported that anti-IL-13 antibodies improved the annual asthma exacerbation rate at week 52 [100]. However, the results of phase 3 studies were not satisfactory [101,102,103]. In those trials, anti-IL-13 antibodies had no benefit for reducing asthma exacerbation and sparing steroid intake.

Another key mediator of type-2 asthma is prostaglandin D2 (PGD2), an upstream mediator of T2 inflammation. PGD2 is mainly produced by mast cells [104]. Fevipiprant, which targets PGD2, is being investigated in phase 3 trials; however, it showed a poor improvement in the FEV1 and no significant reduction in the AER [105,106].

IL-13-dependent chemokines may be involved in severe asthma. CCL-26/eotaxin-3 is important for the migration of eosinophils from the blood to tissues and an approach that blocks CCL-26/eotaxin-3 might reduce eosinophil numbers in the lungs [57,107]. CCL17 and CCL22 chemokines, secreted by DCs, interact with CCR4 receptors expressed by mature Th2 lymphocytes, thus promoting their migration from thoracic lymph nodes to the airways [108]. Because these chemokines and chemokine receptors are involved in the signal cascade of asthma, they may be candidate therapeutic targets for severe asthma in the future.

Regarding therapeutic antibodies, attempts to generate bispecific antibodies targeting more than one cell or receptor are also being tested. Bispecific antibodies have become of increasing interest as therapeutic agents for asthma. Bispecific antibodies can be directed against different signaling pathways simultaneously by binding to two different targets, thus enhancing drug delivery. Compared with combination therapy using two monospecific agents, the use of bispecific antibodies can reduce the cost of development and clinical trials. A bispecific antibody targeting IL-4Ra/IL-5 is under preclinical investigation [109]. The monovalent bispecific antibody Zweimabs and the bivalent bispecific Doppelmab against TSLP/IL-13 have been developed to target Th2 responses [110]. These bispecific antibodies have a strong affinity for human target molecules compared with the parental antibody formats, but with comparable effects. An anti-IL-13/IL-17 antibody (BITS7201A), which can be used for mix-typed eosinophilic and neutrophilic inflammation, is being investigated in a phase I trial [111].

**Figure 3:**
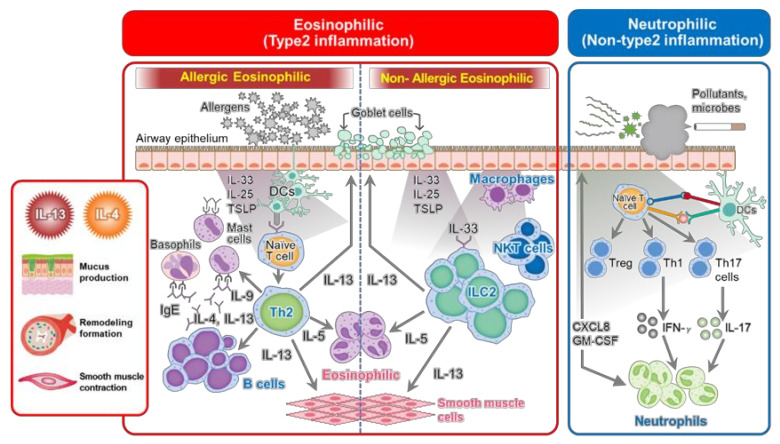
Schematic of the diverse signaling pathways determining bronchial asthma onset and development.

As described previously, mediators of Th2-dependent reactions have a key role in the pathogenesis of asthma. However, non-Th2 dominant type patients also exist, and the regulators of non-type 2 inflammation in asthma include Th17 cells and neutrophils (Figure 3). Th17 cells secrete proinflammatory cytokines such as IL-17A and IL-17F. IL-17A was elevated in the sputum of asthmatic patients and correlated with IL-8, neutrophils in the sputum, and asthma severity [11]. Therefore, asthma is a heterogeneous chronic inflammation with different pathophysiologies, and crosstalk between each cascade augments its severity and increases its intractability. Therefore, treatment strategies targeting non-Th2 type asthmatic components are urgently needed.

Bronchial thermoplasty (BT), a non-pharmacological treatment developed and performed worldwide for severe asthma, alleviates the symptoms of asthma patients. Bronchial smooth muscle is thickened by applying high-frequency energy from a probe inserted transbronchoscopically to the airway wall. It aims to reduce asthma attacks by reducing smooth muscle contractility. In the AIR2 Trial, a large randomized, double-blind study, BT improved the Asthma Quality of Life Questionnaire (AQLQ) and reduced the frequency of exacerbations in severe asthma patients [112]. These effects persisted for 5 years after treatment [113], indicating there is a population of severe asthmatics who are highly responsive to BT. However, no improvement in the FEV1 or airway hyperresponsiveness was observed, and no predictive markers for treatment responses have been identified. Although some experts propose that BT should be considered for severe asthma associated with non-type 2 inflammation or patients who fail to respond to biological therapies targeting type 2 inflammation, the position of BT for the management of severe asthma is still unclear [114]. Therefore, BT can only be positioned as a treatment option for asthma.

Regarding the future developments of asthma treatment, it will be important to fully understand the pathogenesis of asthma. Most importantly, biomarkers must be used to identify disease endotypes and to develop more effective therapeutic approaches.

## 7. Conclusions

Considerable advances have been made in antibody therapeutics for severe asthma over the last decade. Increasing numbers of biological therapies will be introduced to clinical settings. Therefore, it will be of great importance for clinicians to consider the target and mechanism of action of each therapeutic antibody when selecting an appropriate treatment option for individual patients with severe asthma. The current selection and treatment strategies of antibody therapeutics are primarily based on Th2 type disease and eosinophilic inflammation. Further studies are necessary to compare the effects of each antibody type and clarify their effects against airway remodeling and hypersensitivity, and to guide decision making regarding appropriate antibody therapeutics by establishing a real phenotype classification that will predict the response of patients to antibody treatments. Importantly, a better understanding of non-Th2 type immunological mechanisms is urgently required to help develop treatment strategies using antibodies against non-Th2 type signaling cascades.

## Figures and Tables

**Table 1:** Current strategies of biological therapies for severe asthma.

Target	Drug Name	Molecular Mechanisms	Pathophysiological Effect	Predictors of Efficacy	Changes in Clinical Parameters
FcεRI- binding domain of IgE	Omalizumab (Genentech/ Novartis)	Inhibit of IgE– mediated cascade	•Airway allergic inflammation •Airway hyperresponsiveness [38] •Airway remodeling [38]	•Specific IgE antibody positivity or skin prick test [39] •Increase of serum IgE at 4 week [40] •High eosinophils (blood) [41] •High FeNO [41]	•Decrease exacerbationrate [42] and FeNO [41] •Increase FEV1 and ACQ [43] •Reduce OCS [43]
IL-5	Mepolizumab (Glaxo Smithline)	Inhibit the activity of IL-5 by preventing IL-5 to bind IL-5R	•Airway eosinophilic inflammation •Airway hyperresponsiveness * [33] •Airway remodeling * [30]	•High eosinophils (sputum, blood) [44,45] • Nasal polyposis [46] • Lower BMI [46] • Lower OCS [46] • Lower CCL4/MIP-1β [47]	•Decrease exacerbation rate and eosinophils [44,45] •Increase FEV1 [45,48] and ACQ [44,45] •Reduce OCS [44]
	Reslizumab (Teva Pharmaceuticals)	Inhibit of IL-5 signaling			
IL-5Rα	Benralizumab (AstraZeneca)	Blockade of IL-5Rα, and ADCC-induced eosinophil apoptosis	•Airway eosinophilic inflammation •Airway hyperresponsiveness * [33] •Airway remodeling * [34]	•High eosinophils (sputum, blood) [49] • Nasal polyposis [49,50] • Low lung function [50] • age at diagnosis ≥18 years [50]	•Decrease exacerbation rate and eosinophils [51] •Increase FEV1 and ACQ [52] •Reduce OCS [52]
IL-4Rα	Dupilumab (Sanofi/ Regeneron)	Dual blockade of IL4/IL-4Rα and IL-13/IL-13Rα binding	Airway inflammation •Airway hyperresponsiveness [53] •Airway remodeling [54]	•High IgE [55] •High eosinophils [55,56] (sputum, blood) •High FeNO [55,56] • Chronic Sinusitis and nasal polyposis [57]	•Decrease circulating IgE, exacerbation rate, and FeNO [55] •Decrease blood eosinophils after transient increase [55] •Increase FEV1 and ACQ [55] •Reduce OCS [55]

FeNO: Fractional exhaled nitric oxide, FEV1: Forced expiratory volume in 1, ACQ: asthma control score, OCS: oral corticosteroids, CCL4: chemokine (C-C motif) ligand 4, MIP-1β: macrophage inflammatory protein-1β. * This was suggested to be effective ex vivo or in animal models.

## Data Availability

The datasets supporting the conclusions of this article are included within the article. The data sets generated and analyzed in this study are available from the corresponding author on reasonable request.

## Abbreviations

ICS	Inhaled corticosteroids
OCS	Oral corticosteroids
SPDEF	SAM pointed domain containing ETS transcription factor
Foxa	Forkhead box protein
TSLP	Thymic stromal lymphopoietin
EMT	Epithelial-mesenchymal transition
TGF	Transforming growth factor
SARP	Severe Asthma Research Program
ILC2	Group 2 innate lymphoid cells
ILC3	Group 2 innate lymphoid cells
BAL	Bronchoalveolar lavage
FeNO	Fractional exhaled nitric oxide
FEV1	Forced expiratory volume in 1 s
ADCC	Antibody-dependent cell-mediated cytotoxicity
BPT	Bronchial provocation test
FEF	Forced expiratory flow
FOT	Forced oscillation technique
BMI	Body mass index
R5	Respiratory reactance at 5 Hz
R20	Respiratory reactance at 20 Hz
DC	Dendritic cell
PGD2	Prostaglandin D2
BT	Bronchial thermoplasty
AQLQ	Asthma Quality of Life Questionnaire
